# Altered expression of DLG1-AS1 distinguished papillary thyroid carcinoma from benign thyroid nodules

**DOI:** 10.1186/s12902-019-0440-x

**Published:** 2019-11-12

**Authors:** Tao He, Huan Wang, Jiangming Sun, Jie Wu, Fakuo Gong, Shujun Li, Hui Wang, Yufeng Li

**Affiliations:** 1 Department of Nuclear Medicine, Panzhihua Central Hospital, Sichuan Province 617067 Panzhihua City, People’s Republic of China; 2 Department of Ultrasound, Panzhihua Central Hospital, No. 34 YiKang street, Eastern District, Sichuan Province 617067 Panzhihua City, People’s Republic of China

**Keywords:** DLG1-AS1, Papillary thyroid carcinoma, Benign thyroid nodules, miR-199a-3p, Proliferation

## Abstract

**Background:**

Benign thyroid nodules (BTN) are frequently diagnosed as papillary thyroid carcinoma (PTC), leading to unnecessary treatment. We found that plasma lncRNA DLG1-AS1 was upregulated in PTC patients but not in BTN patients and healthy controls.

**Methods:**

In this study DLG1-AS1 and miR-199a-3p in plasma of both PTC patients and BTN patients were detected by qPCR. ROC curve analysis was performed for diagnostic analysis. Overexpression experiments were performed to analyze the interaction between DLG1-AS1 and miR-199a-3p. CCK-8 assay was performed to analyze cell proliferation.

**Results:**

In this study, upregulation of DLG1-AS1 distinguished PTC patients from BTN patients and healthy controls. Plasma miR-199a-3p was downregulated in PTC patients compared with healthy controls and BTN patients. Plasma levels of miR-199a-3p were inversely correlated in PTC patients, but not in BTN patients and healthy controls. miR-199a-3p overexpression failed to significantly affect DLG1-AS1, while DLG1-AS1 overexpression resulted in downregulated miR-199a-3p, In addition, DLG1-AS1 overexpression promoted the proliferation of PTC cells. miR-199a-3p overexpression played an opposite role and attenuated the effects of DLG1-AS1 overexpression.

**Conclusions:**

Therefore, DLG1-AS1 may promote PTC by downregulating miR-199a-3p.

## Background

In 2018, thyroid cancer affected 130,889 new cases in males and 436,344 new cases in females, and the incidence rate in females is more than 3 times higher than in males [1]. Papillary thyroid carcinoma (PTC) is the most common type of thyroid cancer [2]. PTC is a type of slow-growing, well-differentiated, and localized thyroid cancer, although it can metastasize [2]. Comparing to other malignancies mortality rate of thyroid cancer is relatively low, which is mainly due to its less aggressive nature and slow development [3]. In 2018, thyroid cancer only caused 41,000 deaths, which is only about 7% of the newly diagnosed cases [1]. However, metastasis will inevitably occur in some cases of PTC, leading to poor prognosis [4, 5]. Therefore, early diagnosis is still critical. However, PTC in some cases can be misdiagnosed as other thyroid disorders, such as benign thyroid nodules [6]. Therefore, how to distinguish PTC from BTN is a challenge in clinical treatment.

Taking advantage of the non-invasive nature, circulating markers have been widely used for disease prediction [7]. Long no-coding RNAs (> 200 nt, lncRNAs) are RNA transcripts encoding no protein [8]. However, lncRNAs can regulate the expression of protein-coding genes at multiple levels to participate in diverse pathological and physiological processes [8]. Although lncRNAs are usually spatially and/or temporally expressed, they can be released into blood circulation system after synthesis to traffic to other positions of the body [9, 10]. Therefore, the dysregulation of lncRNAs under pathological conditions may be reflected by its levels in plasma [9, 10]. DLG1-AS1 is a recently identified oncogenic lncRNA in cervical cancer [11]. MiR-199a-3p is a well-characterized tumor suppressive miRNA in cancer biology [12–14]. We observed the inverse correlation between DLG1-AS1 and miR-199a-3p from our preliminary bioinformatics analysis. This study aimed to explore the roles of DLG1-AS1 and miR-199a-3p in PTC and analyze the clinical values of DLG1-AS1 for this disease.

## Methods

### Research subjects

Research subjects in this study included 60 PTC patients (16 males and 44 females; age: 29 to 67 years; mean: 48.1 ± 5.5 years), 60 BTN patients (16 males and 44 females; age: 30 to 67 years; mean: 48.3 ± 5.9 years), and 60 healthy controls (16 males and 44 females, age: 28 to 67 years; mean: 47.9 ± 5.0 years). This study passed the review board of Panzhihua Central Hospital. All the participants were selected in Panzhihua Central Hospital during April 2015 and April 2019. PTC and BTN patients were confirmed by histopathological biopsy. Patients’ inclusion criteria: 1) newly diagnosed cases; 2) no therapies were initiated before the admission of patients. Exclusion criteria: 1) recurrent cases; 2) other clinical disorders were observed; 3) history of malignancy; 4) therapies were performed. The 60 PTC patients were at AJCC stage I (*n* = 28) and II (*n* = 32). All participants were educated the principle of experimental designed and informed consent was signed by all of them.

### Blood extraction and plasma preparation

On the day of admission (before the initiation of therapies), fasting blood (5 ml) was extracted from all participants. To separate plasma, blood was injected into EDTA tubes and the tubes were centrifuged for 20 min at 1200 g.

### PTC cells and transient transfections

IHH-4 and MDA-T32 (ATCC, USA) two human PTC cell lines were included. The cell culture medium for both cell lines was prepared by mixing DMEM and FBS with a ratio of 9:1. Cell culture conditions were 95%, 37 °C, and 5% CO_2_. DLG1-AS1 expression vector was constructed using pcDNA3.1 vector (GenePharma, Shanghai, China). Scramble microRNA mimics were designed as negative control microRNA (NC) and miR-199a-3p were both from RIBOBIO77 (Guangzhou, China). At confluence of 70–80, IHH-4 and MDA-T32 cells were harvested and counted, followed by the transfection of 40 nM miRNA (NC miRNA as NC group) or 10 nM vector (empty vector as NC group) into 3× 10^6^ cells through transient transfections mediated by lipofectamine 2000 (Invitrogen, USA). Control (C) cells for all groups were untransfected cells. All subsequent experiments were performed using cells harvested at 24 h post-transfection.

### RNA extraction

Total RNAs in 0.2 ml plasma and 3× 10^5^ cells were extracted using Trizol reagent (Invitrogen, USA). To harvest miRNAs, RNA samples were precipitated and washed using 85% ethanol. All RNA samples were digested with DNase I for 2 h to remove genomic DNA.

### QPCR

AMV reverse transcriptase (GIBCO, USA) was used to transcribe total RNAs into cDNA. Using cDNA as template, FastStart Universal SYBR Green Master (Sigma-Aldrich) was used to prepare qPCR mixtures to measure the expression levels of DLG1-AS1. To measure the expression levels of miR-199a-3p, All-in-One™ miRNA qRT-PCR Reagent Kit (Genecopoeia) was used to perform all steps, including 1′ polyadenylation, reverse transcription and qPCR assays (U6 as endogenous control). 2^**-ΔΔCT**^ method was used for data normalizations and three replicates were included in each experiment.

### Cell proliferation assay

IHH-4 and MDA-T32 cells were harvested at 24 h post-transfection. Cells were counted and 1 ml DMEM (10% FBS) was used to resuspend cell pellets (3× 10^4^ cells) to make single cell suspensions. A 96-well cell plate was used to cultivate cells (0.1 ml per well) under the aforementioned conditions. CCK-8 solution (10 μl, Sigma-Aldrich) was added into each well at 4 h before the end of cell culture. After cell culture was terminated, 10 μl DMSO was added into each well and OD values at 450 nm were measured.

### Statistical analysis

Experiments were performed in 3 biological replicates and data were expressed as mean values. ANOVA (one-way) in combination with Tukey test were used to explore differences among different groups of participants or among different cell transfection groups. Correlation analyses were performed using linear regression. Diagnostic analyses were performed using ROC curve analysis. In ROC curve analysis, BTN patients or healthy controls were true negative cases, and PTC patients were true positive cases. *P* < 0.05 was statistically significant.

## Results

### Plasma DLG1-AS1 was upregulated in PTC patients

**Fig. 1:**
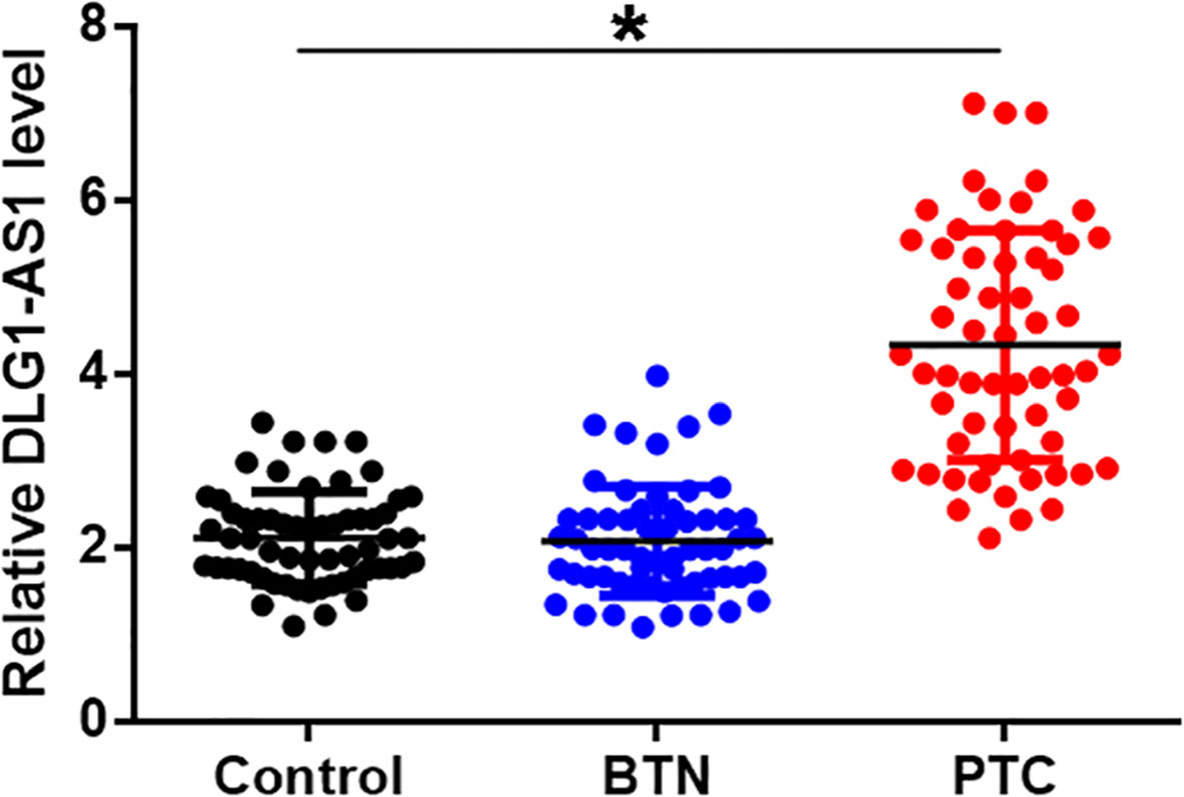
Plasma DLG1-AS1 was upregulated in PTC patients. Levels of DLG1-AS1 in plasma derived from 3 groups of participants (PTC, BTN and Control) were measured and compared by QPCR and ANOVA (one-way) in combination with Tukey test, respectively. Data were expressed as mean values of 3 replicates, *, *p* < 0.05

Levels of DLG1-AS1 in plasma derived from 3 groups of participants (PTC, BTN and Control) were measured and compared by QPCR and ANOVA (one-way) in combination with Tukey test, respectively. Comparing to Control group, significantly higher plasma levels of DLG1-AS1 were found in PTC patients but not in BTN patients (Fig. 1, *p* < 0.05).

### Plasma DLG1-AS1 had diagnostic potentials for PTC

**Fig. 2:**
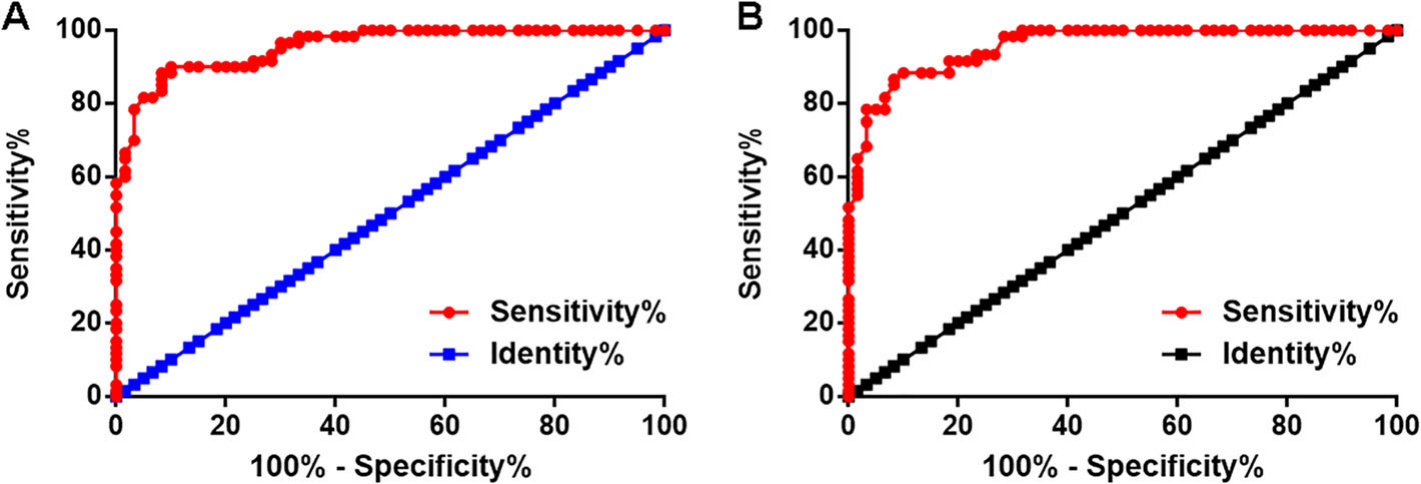
Plasma DLG1-AS1 had diagnostic potentials for PTC. Diagnostic values of DLG1-AS1 for PTC were analyzed using ROC curve. In ROC curve, BTN patients (**a**) or healthy controls (**b**) were true negative cases, and PTC patients were true positive cases

Diagnostic values of DLG1-AS1 for PTC were analyzed using ROC curve through aforementioned methods (Fig. 2). Area under the curve (AUC) > 0.65 indicates diagnostic potentials. With BTN as true negative cases, AUC was 0.95 (95% confidence interval: 0.92–0.99; standard error: 0.016). With healthy controls as true negative cases, AUC was 0.96 (95% confidence interval: 0.93–0.99; standard error: 0.016).

### Plasma miR-199a-3p was specifically downregulated in PTC and was positively correlated with DLG1-AS1 in PTC

**Fig. 3:**
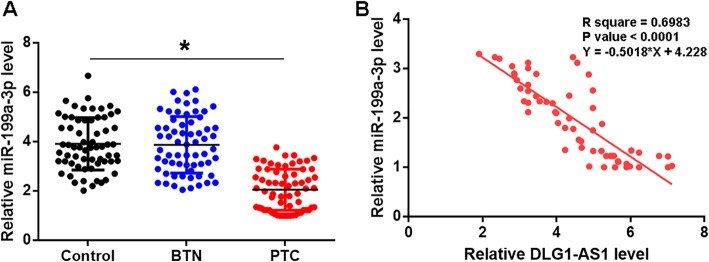
Plasma miR-199a-3p was specifically downregulated in PTC and was positively correlated with DLG1-AS1 in PTC. Levels of miR-199a-3p in plasma derived from 3 groups of participants (PTC, BTN and Control) were measured and compared by QPCR and ANOVA (one-way) in combination with Tukey test, respectively (**a**). The correlation between miR-199a-3p and DLG1-AS1 in PTC patients was analyzed by linear regression (**b**). Data were expressed as mean values of 3 replicates, *, *p* < 0.05

Levels of miR-199a-3p in plasma derived from 3 groups of participants (PTC, BTN and Control) were measured and compared by QPCR and ANOVA (one-way) in combination with Tukey test, respectively. Comparing to Control group, significantly lower plasma levels of miR-199a-3p were found in PTC patients but not in BTN patients (Fig. 3a, *p* < 0.05). The correlation between miR-199a-3p and DLG1-AS1 was analyzed by linear regression. In PTC patients, plasma levels of DLG1-AS1 were significantly and inversely correlated with the expression levels of miR-199a-3p (Fig. 3b). However, the correlation between miR-199a-3p and DLG1-AS1 was not significant in BTN patients and heathy controls (data not shown).

### DLG1-AS1 downregulated miR-199a-3p in IHH-4 and MDA-T32 cells

**Fig. 4:**
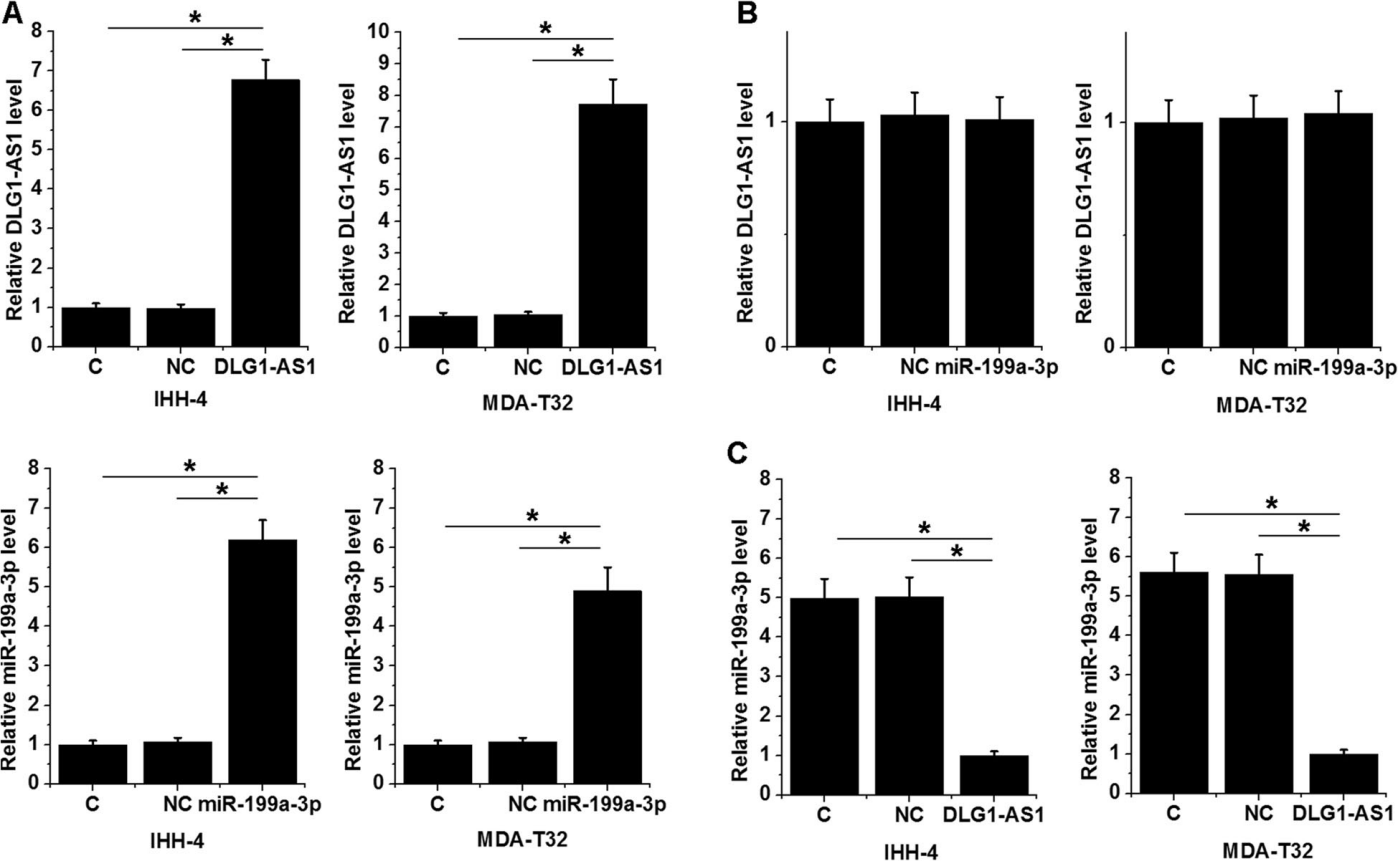
DLG1-AS1 downregulated miR-199a-3p in IHH-4 and MDA-T32 cells. IHH-4 and MDA-T32 cells were transfected with DLG1-AS1 expression vector or miR-199a-3p mimic. Overexpression of DLG1-AS1 and miR-199a-3p was confirmed by qPCR (**a**). The effects of miR-199a-3p overexpression on DLG1-AS1 expression (**b**) and the effects of DLG1-AS1 overexpression on miR-199a-3p expression (**c**) were also analyzed by qPCR. Data were expressed as mean values of 3 replicates, *, p < 0.05

IHH-4 and MDA-T32 cells were transfected with DLG1-AS1 expression vector or miR-199a-3p mimic. Expression levels of DLG1-AS1 and miR-199a-3p were measured by qPCR. Comparing to NC and C two groups, expression levels of DLG1-AS1 and miR-199a-3p were significantly upregulated (Fig. 4a, *p* < 0.05). Moreover, miR-199a-3p overexpression failed to significantly affect DLG1-AS1 (Fig. 4b), while DLG1-AS1 overexpression resulted in downregulated miR-199a-3p (Fig. 4c, *p* < 0.05).

### DLG1-AS1 promoted PTC cell proliferation through miR-199a-3p

**Fig. 5:**
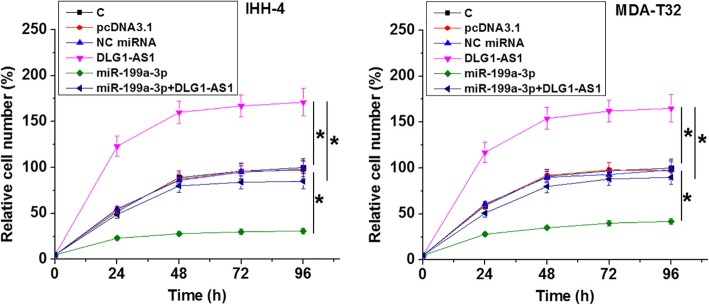
DLG1-AS1 promoted PTC cell proliferation through miR-199a-3p. The effects of DLG1-AS1 and miR-199a-3p overexpression on the proliferation of IHH-4 (**a**) and MDA-T32 (**b**) cells were analyzed by CCK-8 assay. Data were expressed as mean values of 3 replicates, *, *p* < 0.05

The effects of DLG1-AS1 and miR-199a-3p overexpression on the proliferation of IHH-4 (Fig. 5a) and MDA-T32 (Fig. 5b) cells were analyzed by CCK-8 assay. Comparing to C and NC groups, DLG1-AS1 overexpression promoted the proliferation of PTC cells. MiR-199a-3p overexpression played an opposite role and attenuated the effects of DLG1-AS1 overexpression (*p* < 0.05).

## Discussion

This study mainly investigated the roles of DLG1-AS1 in PTC. We found that DLG1-AS1 was upregulated in plasma of PTC patients, and DLG1-AS1 overexpression in PTC cells may downregulate miR-199a-3p to promote the proliferation of PTC cells.

The expression pattern and function of DLG1-AS1 have been investigated in cervical cancer [11]. In cervical cancer, DLG1-AS1 was overexpressed and upregulation of DLG1-AS1 led to the accelerated progression of disease [11]. However, in that study the expression of DLG1-AS1 has only been investigated in tumor and adjacent non-tumor tissues, while the existence of circulating DLG1-AS1 is unknown. In this study we detected the existence of DLG1-AS1 in plasma of all participants. It is known that circulating lncRNAs may serve as systemic signaling to regulate the expression of genes [15]. Therefore, DLG1-AS1 may also regulate gene expression in distant targets after trafficking in blood. Our future studies will explore this possibility.

PTC shares similar imaging features with BTN [16]. Therefore, in clinical practices PTC can be misdiagnosed as BTN, which leads to delayed treatment. Therefore, molecular profiling has been developed to distinguish PTC from BTN [17]. In this study we found that increased plasma levels of DLG1-AS1 can be used to separate early stage PTC patients (stage I and II) from healthy controls and BTN patients. Therefore, measurement of the levels of circulating DLG1-AS1 may assist the early diagnosis of PTC patients, thereby benefitting the survival of PTC patients. We will in future studies include more patients to further analyze the accuracy.

MiR-199a-3p is a well-characterized tumor suppressive miRNA in several types of cancer [12–114]. In hepatocellular carcinoma, miR-199a-3p suppresses cancer cell proliferation by targeting CD44 [12]. In gastric cancer, miR-199a-3p inhibits cancer progression by targeting oncogenic ZHX1 [113]. In a recent study, Minna el at also showed the tumor suppressive role of miR-199a-3p in PTC [14]. In this study we also reported the downregulation of miR-199a-3p in PTC and the increased rate of PTC cell proliferation after miR-199a-3p overexpression. Our data confirmed the tumor suppressive role of miR-199a-3p in PTC. Our study also showed that DLG1-AS1 could downregulate miR-199a-3p to promote cancer cell proliferation. However, the mechanism of the interaction between miR-199a-3p and DLG1-AS1 is unclear. Our future studies will perform deeper investigations.

This study only included Han Chinses, which may provide biased results. The values of DLG1-AS1 in the diagnosis of PTC among other populations remain to be further explored.

## Conclusions

In conclusion, DLG1-AS1 plays oncogenic roles in PTC by downregulating miR-199a-3p to promote cancer cell proliferation. In addition, measurement of the levels of DLG1-AS1 in plasma among the population with high risk of PTC may improve the early diagnosis.

## Data Availability

The analyzed data sets generated during the study are available from the corresponding author on reasonable request.

## Abbreviations

> 200 nt, lncRNAs: Long no-coding RNAs
BTN: Benign thyroid nodules
PTC: Papillary thyroid carcinoma
